# Pilot Study Comparing the Ocular Deviation Measures of Basic Intermittent Exotropia Using 2WIN-S and Prism Cover Test

**DOI:** 10.22599/bioj.534

**Published:** 2025-12-26

**Authors:** Basanta Singh, Bijay Khatri, Rinkal Suwal

**Affiliations:** 1 Department of Optometry, B.P. Eye Foundation, Hospital for Children, Eye, ENT, and Rehabilitation Services, Madhyapur Thimi, Bhaktapur, Nepal; 2 Research Unit for Global Health Department of Public Health, Aarhus University, Aarhus C, Denmark; 3 Centre for Vision and Eye Research, Optometry and Vision Science, Queensland University of Technology, Brisbane, Queensland, Australia

**Keywords:** photorefractometer, intermittent exotopia, basic-type intermittent exotropia

## Abstract

**Introduction:**

Intermittent exotropia (IXT) is a common form of strabismus in children, but the fluctuating angle of deviation and control make measuring the ocular deviation complicated. The prism cover test (PCT) is the gold standard, yet examiner dependent. We assessed whether the 2WIN-S photoscreener (2WIN plus Kaleidos corneal-reflex wand) can quantify deviation in basic IXT and whether control level modifies agreement with PCT.

**Methods:**

Nineteen children with basic-type IXT and monocular acuity 0.1 log MAR or better were enrolled, ocular pathology or significant refractive error excluded. Control was graded (0–2 good; 3–5 fair-to-poor). PCT measured deviation at 4m, 1m and 40 cm with 5 s occlusion; 2WIN-S measured at 1 m after a 30 min rest. Analyses compared 10–20 versus > 20 prism dioptres (pd) and used Bland-Altman plots.

**Results:**

Mean age was 10.26 ± 3.76 years. Median deviation was 25.0 pd (IQR 20–30) with PCT versus 8.5 pd (IQR 0–19.5) with 2WIN-S (z = –3.82, p < 0.001). Differences were significant in both 10–20 pd (median 11.25; z = –2.20, p = 0.028) and > 20 pd groups (median 18.0; z = –3.18, p = 0.001). In five cases, 2WIN-S reported orthophoria; four had ≥ 20 pd on PCT and good control. Good-control cases showed a 20.0 pd median difference; fair-to-poor control showed 3.5 pd within the 10 pd clinical margin. Bland-Altman mean difference was 14.13 pd with limits of agreement from –4.96 to 33.22.

**Conclusion:**

Overall agreement between 2WIN-S and PCT was poor. Until methodological or optical refinements are made, 2WIN-S should not be used for quantifying basic-type IXT.

## Introduction

Intermittent exotropia (IXT) is an outward ocular deviation that intermittently breaks fusional control, producing manifest exotropia when control lapses (Rutstein and Corliss, 2003). It is one of the most common forms of strabismus, with a prevalence of about 1% in children (Bruce and Santorelli, 2016; Chia *et al.*, 2010; Yu *et al.*, 2002; Govindan *et al.*, 2005). Because binocular single vision in IXT is sustained only through extra convergence and accommodation, patients fatigue quickly, and bright light can overload retinal fusion mechanisms, precipitating tropic episodes (Hirota *et al.*, 2020; Westman and Liinamaa, 2012; Figueira and Hing, 2006; Oh, Jung and Shin, 2023). These frequent losses of alignment diminish self-esteem and overall quality of life for both children and their families (Oh, Jung and Shin, 2023; Satterfield, Keltner and Morrison, 1993), and in some cases, ultimately progress to constant exotropia (Rutstein and Corliss, 2003).

Clinical evaluation and management of IXT are challenging. Onset is often within the first year of life (Costenbader, 1950), yet its aetiology remains multifactorial—innervational imbalance, mechanical factors, fusion capacity, AC/A ratio anomalies, refractive errors, and hemiretinal suppression have all been implicated (Lee *et al.*, 2018; Wu *et al.*, 2021; Bui Quoc and Milleret, 2014; Cooper, 1977; Kushner and Morton, 1998; Kekunnaya, Chandrasekharan and Sachdeva, 2015; Serrano-Pedraza *et al.*, 2011). In IXT, whether deviation severity naturally worsens is unclear; however, deterioration is commonly judged by declining ‘control’, which refers to the patient’s ability to regain alignment. Poor control is regarded as a marker of disease progression and may indicate the need for intervention (Rosenbaum and Stathacopoulos, 1992); however, control can fluctuate within minutes (Hatt *et al.*, 2008), so a single assessment is unreliable.

Quantifying deviation magnitude is therefore essential. The prism-cover test (PCT) is the accepted gold standard, yet inter-examiner variability—linked to clinician experience, test distance, dissociation time, and target choice—means that only changes ≥ 10 prism dioptres (pd) are deemed clinically meaningful (Gao *et al.*, 2019; de Jongh *et al.*, 2014; Holmes, Leske and Hohberger, 2008). Hand-held photorefractometers offer a rapid alternative. Devices such as Plusoptix, MTI and 2WIN simultaneously measure refraction and detect strabismus by analysing corneal-reflex position (Schimitzek and Lagrèze, 2005; Saber Moghadam, Alizadeh and Zarei-Ghanavati, 2013; Weinand, Gräf and Demming, 1998; Arnold *et al.*, 2019; Cheng *et al.*, 2021). The latest model, 2WIN-S (Adaptica, Italy), couples the 2WIN camera with a Kaleidos housing and an infrared corneal-reflex (CR) wand that dissociates the eyes while blocking visible light (Suwal *et al.*, 2023). Kaleidos is a rectangular tube that holds the 2WIN with an added battery on one end, and features a dark tunnel on the other to maintain the ideal viewing distance, promote steady fixation, and induce natural pupil dilation (Martin *et al.*, 2020). This design permits measurement in bright ambient conditions and at a fixed viewing distance of ~1 metre.

Although photorefractometers have been validated for constant exotropia and other deviations (Arnold *et al.*, 2019; Arthur *et al.*, 2009), no study has examined how control level affects measurement accuracy in IXT, and 2WIN-S provides no dedicated IXT output. The present pilot study, therefore, compares the deviation magnitude recorded by 2WIN-S with that obtained by the gold-standard PCT in basic-type IXT and investigates whether good versus fair-to-poor control influences agreement between the two methods.

## Methods

This clinical pilot comparative study was approved by the Ethical Review Board, Nepal Health Research Council. The study followed the tenets of the Declaration of Helsinki. Written assent was obtained from the children, and their legal guardians provided written consent at the Hospital for Children, Eye, ENT, and Rehabilitation Services. The study was conducted from January to December 2023.

Nineteen participants (six males, 13 females) with a history of IXT were enrolled. The inclusion was limited to participants with monocular visual acuity of 0.1 log MAR or better in both eyes. Participants with basic IXT—showing equal angles of exodeviation at distance and near—were included to ensure a homogeneous sample and reduce variability. The PCT was performed at 4 metres, 1 metre, and 40 cm, and only those demonstrating equal deviation magnitudes across all three distances and equal office-based control scores for distance and near were selected. As the 2WIN-S measures deviation at approximately 1 metre (matching Kaleidos’ length) and does not measure distance deviation (Suwal *et al.*, 2023), ocular deviations measured with 2WIN-S were compared with the 1 metre PCT measurements.

Participants with ocular pathology, refractive error > ± 0.50 D sphere or > ± 0.50 D cylinder, prior ocular surgery, ptosis, or seizure disorder were excluded. Control of basic-type IXT was evaluated for distance and near using an office-based scale ranging from 0 to 5 (Mohney and Holmes, 2006).

- **Levels 0–2** (good control) require brief dissociation:
  - 0 = phoria; misalignment recovers quickly.
  - 1 = eyes drift outward but recover within 1–5 s.
  - 2 = same drift but recovery > 5 s.
- **Levels 3–5** (fair-to-poor control) require a 30 s observation before dissociation:
  - 3 = exotropia < 50% of the time.
  - 4 = exotropia > 50% of the time.
  - 5 = constant exotropia.

Control levels 0–2 were designated as ‘good’ and 3–5 as ‘fair-to-poor’ (Stathacopoulos *et al.*, 1993). Eligible participants were recruited from the Orthoptic Department. Since PCT breaks fusion and control, subjects rested for 30 minutes before 2WIN-S measurements to avoid bias (Anderson *et al.*, 2010). Deviations were also analysed separately for 10–20 pd Base In (BI) and > 20 pd BI groups (measured with PCT).

### Strabismus measurement by PCT

An optometrist performed PCT, covering each eye for five seconds to break fusion at distance (4 m) and near (40 cm and 1 m) (Barnard, 2008). During neutralisation, the eyes were alternately covered several times, typically requiring 10–15 alternations. Subjects with good-control IXT required fewer alternations compared to those with poor control (Hatt *et al.*, 2008). A 0.2 log MAR line at 4 m served as the distance target; a Bernell fixation wand served as the non-accommodative target at 40 cm and 1 m. All measurements were recorded in the primary position.

### Strabismus measurement by 2WIN-S

**Figure 1:**
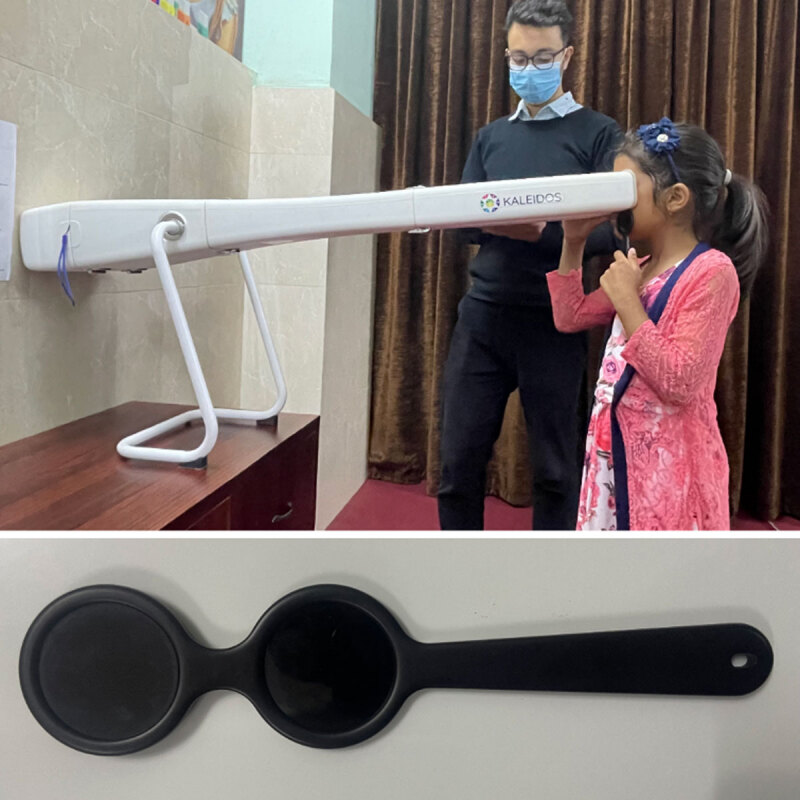
Measurement of ocular deviation with 2WIN-S using infrared light passing CR wand. (Parental permission was given to share the photograph for educational and research purposes).

2WINS (5.5.0) was placed on a table; participants stood or sat so they viewed the target in primary position (Figure 1). Each assessment comprised two monocular and one binocular acquisition. The CR wand was held vertically—right hand for right eye, left hand for left—with the handle down and the edge against the nose. Subjects fixated on the white LED in 2WIN-S. Deviation was calculated from the average corneal-reflex location. Output categories included ET (esotropia), XT (exotropia), EP (esophoria), XP (exophoria), HT (hypertropia), IT (ipotropia), HP (hyperphoria), IP (ipophoria), and ortho, plus deviation magnitude (Adaptica S.r.l, 2021). Data were stored on a Samsung Galaxy Tab A (Android 9) via Bluetooth.

Data analysis was carried out using IBM SPSS 23.0 (IBM Corp., Armonk, NY, USA). Since the data were not normally distributed, Wilcoxon signed-rank test was used to compare the magnitude of the deviation of IXT measured with PCT and 2WIN-S. Bland-Altman plot was used to show the agreement between two measurements methods.

## Results

The deviation measured by PCT and 2WIN-S of each participant and the demographic features of participants in this study were summarised in Tables 1 and 2. The mean age of the participants was 10.26 ± 3.76 years (range 5–16 years). Out of 19 participants, six had a deviation between 10–20 pd BI (range 6–16 years), and 13 had a deviation more than 20 pd BI (range 5–16 years) when measured from PCT.

**Table 1:** Deviation measured by PCT and 2WIN-S of each participant.


S NO.	AGE	GENDER	OCULAR DEVIATION WITH PCT (IN PD BI)	OFFICE-BASED SCALE CONTROL	OFFICE CONTROL	OCULAR DEVIATION WITH 2WIN-S (IN PD)	OUTPUT BY 2WIN-S

1	6	M	16	1	Good	6	Exotropia

2	11	F	18	1	Good	0	Orthophoria

3	7	M	18	1	Good	5.5	Exotropia

4	11	F	20	1	Good	0	Orthophoria

5	13	F	25	1	Good	0	Orthophoria

6	5	F	25	2	Good	6.5	Exotropia

7	8	M	25	1	Good	0	Orthophoria

8	12	F	30	2	Good	12	Exophoria

9	14	F	30	2	Good	0	Orthophoria

10	16	F	30	2	Good	8.5	Exophoria

11	7	M	35	2	Good	5.5	Exotropia

12	16	F	20	3	Fair-to-poor	12	Exophoria

13	6	F	20	3	Fair-to-poor	19.5	Exotropia

14	15	F	25	4	Fair-to-poor	23	Exotropia

15	15	M	25	3	Fair-to-poor	18	Exotropia

16	8	F	25	3	Fair-to-poor	10	Exophoria

17	11	F	25	4	Fair-to-poor	24	Exophoria

18	6	F	25	3	Fair-to-poor	23	Exotropia

19	8	M	30	3	Fair-to-poor	25	Exotropia


PCT: prism cover test; BI: Base In.

**Table 2:** Demographic features of participants.


DEMOGRAPHIC CHARACTERISTICS	BASIC TYPE INTERMITTENT EXOTROPIA				

	DEVIATION		CONTROL		OVERALL PARTICIPANTS

	10–20 PRISM DIOPTRES BI	GREATER THAN 20 PRISM DIOPTRES BI	GOOD CONTROL	FAIR-TO-POOR CONTROL	

Participants number	6	13	11	8	19

Mean age ± SD (95% CI) (years)	9.5 ± 3.93 (95% CI 5.36–13.63)	10.61 ± 3.79 (95% CI 8.32–12.91)	10.00 ± 3.6 (95% CI 7.57–12.42)	10.62 ± 4.2 (95% CI 7.1–14.1)	10.26 ± 3.76 (95% CI 8.44–12.07)


BI: Base In.

Among the 19 participants, the 2WIN-S outputted nine participants with exotropia, five with exophoria, and five with orthophoria. Among the five cases outputted as orthophoria by the 2WIN-S, four participants had IXT with deviation ≥ 20 pd BI (when measured with PCT), and all of them had good control of their deviation with the office-based control scale.

**Figure 2:**
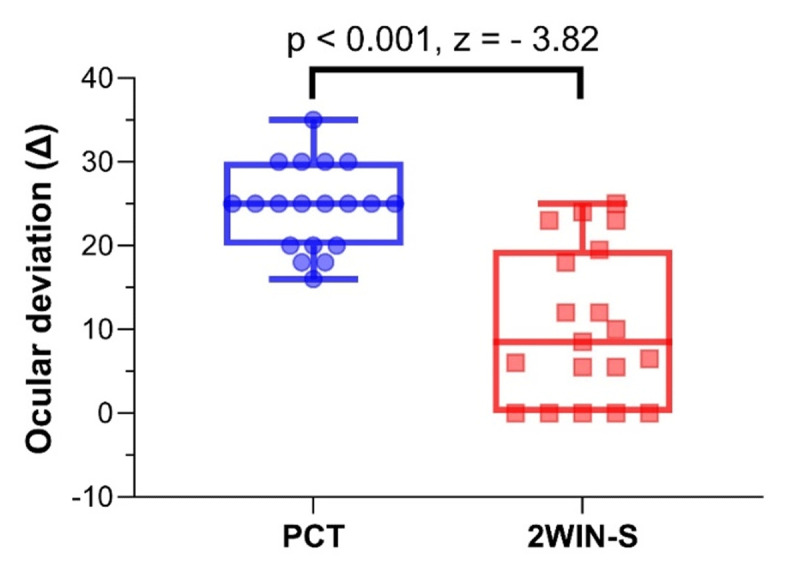
Box and Whisker plot depicting ocular deviation compared with the PCT and 2WIN-S of all participants.

Comparison of deviation between 2WIN-S and PCT was shown in Table 3. Significant differences were observed in the median magnitude measurements between PCT and 2WIN-S, as illustrated in Figure 2 (z = –3.82, p < 0.001). The overall median magnitude for all participants was 25.0 pd (interquartile range [IQR] = 20.0–30.0) when measured with PCT, and 8.50 pd (IQR = 0.0–19.5) with 2WIN-S. Therefore, the 2WIN-S consistently underestimated the magnitude of IXT compared with the PCT. Among participants with IXT between 10 and 20 pd, the mean difference between the two methods was 11.25 pd (IQR = 6.3 to 18.50), a statistically significant finding (z = –2.20, p = 0.028). For those with deviations exceeding 20 pd, the mean difference was 18.0 pd (IQR = 3.50–25.0), also significantly different (z = –3.18, p = 0.001).

**Table 3:** Comparison of the magnitude of strabismus measured with the PCT and 2WIN-S.


PARTICIPANTS	THE MAGNITUDE OF STRABISMUS (MEDIAN (INTERQUARTILE RANGE))			

	PRISM COVER TEST (IN PD BI) MEDIAN (IQR)	CR 2WIN-S (IN PD) MEDIAN (IQR)	DIFFERENCE (IN PD) MEDIAN (IQR)	P-VALUE

Overall participants	25.00 (20.00–30.00)	8.50 (0.00–19.50)	15.00 (5.00–21.50)	< 0.001* (z = –3.82)

Intermittent exotropia Deviation 10–20 prism dioptres	19.00 (17.50–20.00)	5.75 (0.00–13.88)	11.25 (6.13–18.50)	0.028* (z = –2.20)

Intermittent exotropia Deviation greater than 20 prism dioptres	25.00 (25.00–30.00)	10.00 (2.75–23.00)	18.00 (3.50–25.00)	0.001* (z = –3.18

Good control	25.00 (18.00–30.00)	5.50 (0.00–6.50)	20.00 (18.00–25.00)	0.003* (z = –2.94)

Fair-to-poor control	25.00 (21.25–25.00)	21.25 (13.50–23.75)	3.50 (1.25–7.75)	0.012* (z = –2.52)


IQR: Inter Quartile Range; pd: prism dioptre; BI: Base In; * Wilcoxon signed-rank test.

Participants exhibiting good control of IXT had a median difference of 20.0 pd (IQR = 18.0 to 25.0) between PCT and 2WIN-S, which showed a significant difference in magnitude (z = –2.94, p = 0.003). For participants with fair-to-poor control of IXT, the median difference was only 3.50 pd (IQR = 1.25 to 7.75), which was also significantly different (z = –2.52, p = 0.012) but clinically acceptable as the difference was below 10 pd (de Jongh *et al.*, 2014; Holmes, Leske and Hohberger, 2008).

**Figure 3:**
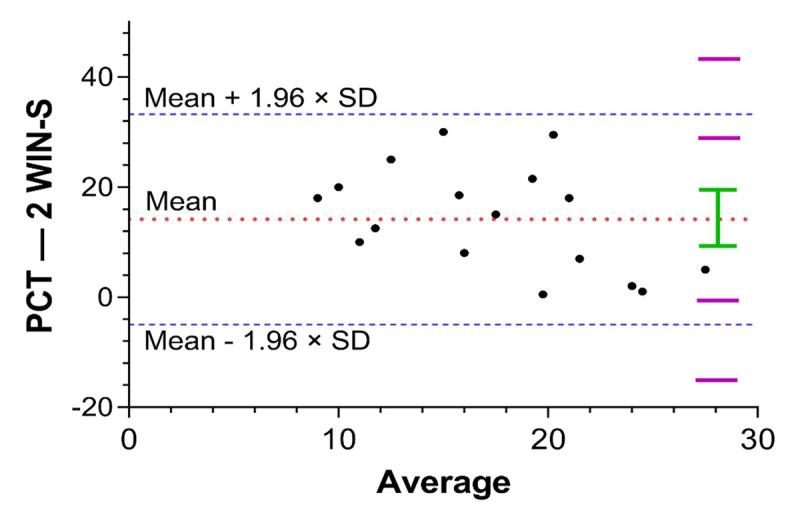
Bland-Altman plot of magnitude in cases of IXT between PCT and 2WIN-S. Mean difference is illustrated by a dotted red line; upper and lower 95% limits of agreement between the two measurements are represented by dotted blue lines and included in the standard deviation of ± 1.96.

Bland-Altman analysis showed that the 95% limits of agreement between PCT and 2WIN-S were 33.22 pd to –4.96 pd (Figure 3). Although all the points were scattered equally between upper and lower limits of agreement, the mean difference of 14.13 pd signifies a lack of agreement between the two methods, as any difference exceeding 10 pd between two measurements is considered as a significant change (Holmes, Leske and Hohberger, 2008; de Jongh *et al.*, 2014).

## Discussion

Quick and accurate measurement of IXT in children is challenging. This study reflects a comparison of ocular deviation between PCT and 2WIN-S measurement in cases with basic type IXT. The findings of this study depict that in cases with fair-to-poor control, basic type IXT, there are clinically acceptable, comparable ocular deviation measurements between 2WIN-S and PCT. However, for cases with good control in basic type IXT, 2WIN-S significance is limited.

The infrared occluder used by 2WIN is an optical cast infrared long-pass filter exhibiting over 90% transmission, having a density of 1.320 g cm^3^, refractive index 1.501, thickness 1.5 mm, and an Abbe value of 57 (Edmund Optics, 2023). The occluder only permits the transmission of light with a wavelength exceeding 750 nm. Since the LED lights of 2WIN-S span 850–870 nm, any deviation under the occluder is detected by the 2WIN-S. However, as infrared light begins at 780 nm (Tsai and Hamblin, 2017), any light between 750 nm and 780 nm can pass through the occluder, so bright light can appear with a pink tint through the filter (Arnold *et al.*, 2019). A similar occluder, the HOYA R72 filter, used with an infrared camera to measure ocular alignment by blocking light below 720 nm, effectively detected latent components of strabismus, including IXT, in good agreement with PCT (Yang *et al.*, 2013). In this study, the CR wand—which blocks light below 750 nm—surpasses the HOYA R72 specification, yet it still failed to identify all basic-type IXT cases.

An important observation was that many participants with basic type IXT (Participants 2, 4, 5, 7 and 9) who had good control were reported as orthophoric by 2WIN-S. Among these five, four had deviations ≥ 20 pd BI. This suggests the instrument’s failure was linked to control rather than magnitude. A similar limitation was noted when the Plusoptix S04 photoscreener missed well-controlled intermittent exotropia < 20 pd while screening for amblyogenic risk factors (Arthur *et al.*, 2009).

When used without Kaleidos, 2WIN consistently reported constant deviation but needed several seconds to detect intermittent deviations in some cases, with reliable measurements for deviations > 10 pd (Arnold *et al.*, 2019). That study did not evaluate control; our results show that 2WIN-S measures deviation reliably in basic IXT with fair-to-poor control, but inconsistently when control is good. Where 2WIN-S failed to detect misalignment, its recorded deviations underperformed PCT values by at least 15 pd (Participant 9; 30 pd with PCT and ortho with 2WIN-S).

The Pediatric Vision Screener (PVS), designed to detect amblyogenic risk factors, reliably detected all intermittent deviations (Nassif, Piskun and Hunter, 2006). 2WIN-S struggled with well-controlled basic IXT. This could be because the two instruments operate on different principles. The Pediatric Vision Screener employs binocular retinal birefringence scanning to detect strabismus by assessing foveal fixation through analysis of polarisation changes unique to the retinal birefringence pattern of the fovea, whereas 2WIN-S relies on the corneal Hirschberg reflex. The PVS study did not stratify by control, possibly explaining its success (Nassif, Piskun and Hunter, 2006). A hybrid device combining PVS and 2WIN-S principles might better detect IXT.

Deviation increases with longer dissociation, and ≥ 5 s occlusion is needed for full horizontal deviation (Anderson *et al.*, 2010; Barnard and Thomson, 1995). In this current study, although PCT employed 5 s occlusion, 2WIN-S completed measurements in < 5 s. Longer occlusion under the infrared filter might improve 2WIN-S accuracy, but extended occlusion contradicts the rapid-screening goal of photoscreeners. Moreover, alternate occlusion in PCT is more dissociative, often requiring several alternations to fully neutralise the deviation of IXT. This dissociative function is absent in 2WIN-S (Deacon and Gibson, 2001; Hatt *et al.*, 2008).

Deteriorating control of IXT is usually not a good indicator and may indicate surgery (Rosenbaum and Santiago, 1999; Von Noorden and Campos, 2002). Non-surgical options, including vision therapy, can improve control (Heydarian *et al.*, 2020; Ma *et al.*, 2021). Thus, assessing control is crucial. 2WIN-S successfully quantified deviation in cases with fair-to-poor control, aligning with clinical practice where interventions hinge on control. However, accurate magnitude measurement remains vital when surgery is considered (Abroms *et al.*, 2001), and this remains a shortcoming of 2WIN-S that warrants refinement in technique or CR-wand optics.

This pilot study has limitations. The small sample restricts precision, yet it is the first to examine how a photoscreener quantifies basic type IXT by control. Inclusion was limited to basic IXT with equal distance-near deviation and identical control scores. Other IXT types—convergence insufficiency, true divergence excess, pseudo-divergence excess—were excluded. Future research should include all IXT types and varied control levels to further assess 2WIN-S performance.

## Conclusion

In this pilot study of basic-type intermittent exotropia, 2WIN-S showed poor overall agreement with the PCT, with marked underestimation in well-controlled cases. Measurements were clinically acceptable only when the control was fair-to-poor. Until methodological or optical refinements are made in both the 2WIN-S system and the CR wand, 2WIN-S should not be used for quantifying basic-type IXT.
